# Supplementary material for: Stochastic model of contact inhibition and the proliferation of melanoma in situ
Source: Sci Rep. 2017 Aug 14;7:8026. doi: 10.1038/s41598-017-07553-6 (PMC5556068; doi:10.1038/s41598-017-07553-6)
Supplement: Supplementary file 1 — Supplementary Information file [file 41598_2017_7553_MOESM1_ESM.pdf]

Supplementary Information for **Stochastic Model of Contact Inhibition and the proliferation of melanoma *in situ***

Mauro César Cafundó de Morais[1,2], Izabella Stuhl[3,4], Alan Utsuni Sabino[2], Willian Wagner Lautenschlager[2], Alexandre Sarmento Queiroga[1], Tharcisio Citrangulo Tortelli Jr.[1], Roger Chammas[1], Yuri Suhov[5,6], and Alexandre Ferreira Ramos[1,2,*]

[1]Department of Radiology and Oncology, Faculty of Medicine, University of Sao Paulo, Sao Paulo, Brazil
[2]School of Arts, Science and Humanities, University of Sao Paulo, Sao Paulo, Brazil
[3]Math Department, University of Denver
[4]University of Debrecen, Hungary
[5]DPMMS, University of Cambridge, UK
[6]Math Department, Penn State University

This manuscript was compiled: June 3, 2017

The authors would like to provide additional information regarding the analyses discussed in the main text, and to explain the study in greater detail.

The mathematical model of contact inhibition

The mathematical model deals with two particle/cell types, 1 (cancer) and 2 (normal) forming a *configuration* $\mathbf{x}_\Lambda$ over a large square area Λ of a 2-dimensional step-one lattice $\mathbb{Z}^2$. Theoretically, it is convenient to treat $\mathbf{x}_\Lambda$ as a restriction to Λ of a configuration $\mathbf{x}$ given over the whole of $\mathbb{Z}^2$. Formally, $\mathbf{x}$ is characterized by a value $\mathbf{x}(y) = 0, 1, 2$ assigned to each site $y \in \mathbb{Z}^2$: if $\mathbf{x}(y) = 0$, site y is treated as vacant, and if $\mathbf{x}(y) = 1$ or 2, the site is considered as occupied by a cancer or normal cell, respectively. We say that $\mathbf{x}$ is *d-admissible* (or simply *admissible*) if (i) any two sites occupied by normal cells should be at distance greater than 1 (i.e., the minimal allowed distance between them is 2 in the graph metric or $\sqrt{2}$ in the Euclidean metric), and (ii) cancer and normal cells are separated by a distance at least d, in the graph metric. Cancer cells can be at distance 1 or larger. Formally, an admissible configuration obeys an *exclusion principle* with the specified collection of minimal distances $\mathbf{D} = \{D(i,j)\}$ (in the graph metric):

$$\begin{array}{r}\mathrm{dist}\,(y,y') \geq 1,\ \text{if } \mathbf{x}(y)=\mathbf{x}(y')=1, \text{ i.e., } D(1,1)=1,\\ \mathrm{dist}\,(y,y') \geq 2,\ \text{if } \mathbf{x}(y)=\mathbf{x}(y')=2, \text{ i.e., } D(2,2)=2,\\ \mathrm{dist}\,(y,y') \geq d,\ \text{if } \mathbf{x}(y)=1,\ \mathbf{x}(y')=2, \text{ i.e., } D(1,2)=d.\end{array} \tag{1}$$

Parameter d can vary but our theoretical results remain the same for all values of d. (The only part which is sensitive to the choice of d is the degree of closeness of the value r to 1 and the position of the point $P(r)$ (see Assertion (B) in Theorem 1 below).

We study probability distributions (or probability measures) $\mu_\Lambda(\,\cdot\,|\mathbf{y}_{\Lambda^c})$ on $\mathcal{A}_\Lambda(\mathbf{y}_{\Lambda^c})$, the collection of admissible configurations $\mathbf{x}_\Lambda$ over Λ compatible with a given *boundary condition* $\mathbf{y}_{\Lambda^c}$ (in the sense of the above notion of admissibility). We will focus on four specific configurations $\mathbf{y}$: (i) with $\mathbf{y}(x) = 1$ for any $x \in \mathbb{Z}^2$, (ii) with $\mathbf{y}(x) = 2$ for any even site $x \in \mathbb{Z}^2$ and $\mathbf{y}(x) = 0$ for any odd site $x \in \mathbb{Z}^2$, (iii) with $\mathbf{y}(x) = 2$ for any odd site $x \in \mathbb{Z}^2$ and $\mathbf{y}(x) = 0$ for any even site $x \in \mathbb{Z}^2$, (iv) with $\mathbf{y}(x) = 0$ for any $x \in \mathbb{Z}^2$. (A site $x = (\mathrm{x}_1, \mathrm{x}_2) \in \mathbb{Z}^\mathrm{d}$ is called even if the sum $\mathrm{x}_1 + \mathrm{x}_2$ is even and odd if it is odd.) In case (i) the configuration is referred to as $\mathbf{y}^{(1)}$, in case (ii) as $\mathbf{y}^{(\mathrm{EV})}$, in case (iii)

as $\mathbf{y}^{(\mathrm{OD})}$ and in case (iv) as $\mathbf{y}^{(0)}$. The pair $\mathbf{y}^{(\mathrm{EV})}$, $\mathbf{y}^{(\mathrm{OD})}$ represents close-packing configurations of type 2 (here exactly a half of the lattice $\mathbb{Z}^d$ is occupied). Similarly, $\mathbf{y}^{(1)}$ yields a close packing configuration of type 1 (all of $\mathbb{Z}^d$ is occupied) while $\mathbf{y}^{(0)}$ is an empty configuration.

Fix a pair of numbers $p_1, p_2 > 0$ with $p_1 + p_2 < 1$. The pair (p_1, p_2) represents a parameter specifying the probability measure $\mu_\Lambda(\,\cdot\,|\mathbf{y}_{\Lambda^{\complement}})$. The locus of points $(p_1, p_2) \in \mathbb{R}^2$ obeying the above conditions $p_1, p_2 > 0$ and $p_1 + p_2 < 1$ is an (open) straight triangle $\mathbb{T}$ with vertices at $(0,0)$ (the origin), $(1,0)$ and $(0,1)$: this is a parameter space for the family of probability distributions $\mu_\Lambda(\,\cdot\,|\mathbf{y}_{\Lambda^{\complement}})$. The triangle $\mathbb{T}$ is sliced into intervals

$$I_r = \{(p_1, p_2): \; p_1, p_2 > 0, \; p_1 + p_2 = r\}, \;\; 0 < r < 1, \tag{2}$$

which are parallel to the hypotenuse: $\mathbb{T} = \bigcup_{0<r<1} I_r$. Let $\mathbb{P}$ denote a *Bernoulli distribution* where each site $y \in \mathbb{Z}^d$ is vacant with probability $1 - p_1 - p_2$, occupied with type 1 with probability p_1 and occupied with type 2 with probability p_2. Then $\mathbb{P}(\mathcal{A}_\Lambda(\mathbf{y}_{\Lambda^{\complement}})) > 0$; this allows us to consider the conditional distribution

$$\mu_\Lambda(\,\cdot\,|\mathbf{y}_{\Lambda^{\complement}}) = \frac{\mathbb{P}(\,\cdot\, \cap \mathcal{A}_\Lambda(\mathbf{y}_{\Lambda^{\complement}}))}{\mathbb{P}(\mathcal{A}_\Lambda(\mathbf{y}^{\complement}_\Lambda))}. \tag{3}$$

We say that $\mu_\Lambda(\,\cdot\,|\mathbf{y}_{\Lambda^{\complement}})$ is a *Gibbs distribution* in Λ with the collection of exclusion diameters $\mathbf{D}$, parameter (p_1, p_2) and a boundary condition $\mathbf{y}_{\Lambda^{\complement}}$. This distribution is associated with the *grand canonical ensemble*. (In the Mathematical Physics terminology, numbers p_1 and p_2 can be related to *fugacities* of particles of type 1 and 2.)

The existing theory allows us to analyse the limiting picture as $\Lambda \nearrow \mathbb{Z}^2$; see Theorem 1 below. For the concept of a *DLR measure* and an *extreme* DLR measure, cf., e.g., [H.-O. Georgii. *Gibbs measures and phase transitions.* Berlin, New York: De Gryter, 2011, Part III]. The notion of a small perturbation of a probability measure is established in terms of the *Pirogov–Sinai theory*; cf. [Ya.G. Sinai. *Theory of phase transitions: rigorous tresults.* Oxford *et al.*: Pergamon Press, 1982].

The following theorem takes place ([A. Mazel, I. Stuhl, Y. Suhov. Work in progress]):

Theorem 1. *For any $d \geq 1$, given a collection of minimal allowed distances $\mathbf{D} = \{D(i,j)\}$ as in (1), there exist* (a) *a number $q \in (0,1]$ (which turns out to equal 1) and* (b) *for every $r \in (0,1)$ a point $(P(r), r - P(r))$ lying in segment I_r, with the following properties.*

(A) *The limit $\lim\limits_{r \to 1} P(r) = q$.*

(B) *Suppose r is close to 1. Then:*

(B1) *For any pair $(p_1, p_2) \in I_r$ with $p_1 + p_2 = r$ and $p_1 > P(r)$ there is exactly one extremal DLR measure $\mu^{(1)}$. This measure is a small perturbation of the degenerate measure sitting on a single configuration $\mathbf{y}^{(1)}$. Furthermore, if $p_1 < P(r)$ then for all admissible configurations $\mathbf{y}$,*

$$\lim_{\Lambda \nearrow \mathbb{Z}^d} \mu_\Lambda(\,\cdot\,|\mathbf{y}_{\Lambda^{\complement}}) = \mu^{(1)}. \tag{4}$$

(B2) *For any pair $(p_1, p_2) \in I_r$ with $p_1 + p_2 = r$ and $p_1 < P(r)$ there are exactly two extremal DLR measures $\mu^{(\mathrm{EV})}$ and $\mu^{(\mathrm{OD})}$. These measures are space-shifts of each other, and $\mu^{(\mathrm{EV})}$ is a small perturbation of the degenerate measure sitting on a single configuration $\mathbf{y}^{(\mathrm{EV})}$ while $\mu^{(\mathrm{OD})}$ is a small perturbation of the degenerate measure sitting on a single configuration $\mathbf{y}^{(\mathrm{OD})}$. Furthermore, for $p_1 < P(r)$:*

$$\lim_{\Lambda \nearrow \mathbb{Z}^d} \mu_\Lambda(\,\cdot\,|\mathbf{y}^{(\mathrm{EV})}_{\Lambda^{\complement}}) = \mu^{(\mathrm{EV})}, \qquad \lim_{\Lambda \nearrow \mathbb{Z}^d} \mu_\Lambda(\,\cdot\,|\mathbf{y}^{(\mathrm{OD})}_{\Lambda^{\complement}}) = \mu^{(\mathrm{OD})} \tag{5}$$

and

$$\lim_{\Lambda \nearrow \mathbb{Z}^d} \mu_\Lambda(\,\cdot\,|\mathbf{y}^{(1)}_{\Lambda^{\complement}}) = \lim_{\Lambda \nearrow \mathbb{Z}^d} \mu_\Lambda(\,\cdot\,|\mathbf{y}^{(0)}_{\Lambda^{\complement}}) = (\mu^{(\mathrm{EV})} + \mu^{(\mathrm{OD})})/2. \tag{6}$$

(B3) *For any pair $(p_1, p_2) \in I_r$ with $p_1 + p_2 = r$ and $p_1 = P(r)$ there are exactly three extremal DLR measures $\mu^{(\mathrm{EV})}$, $\mu^{(\mathrm{OD})}$ and $\mu^{(1)}$. As before, $\mu^{(\mathrm{EV})}$ and $\mu^{(\mathrm{OD})}$ are space-shifts of each other, and each of $\mu^{(\mathrm{EV})}$, $\mu^{(\mathrm{OD})}$ and $\mu^{(1)}$ is a small perturbation of the degenerate measure sitting on the respective single configuration $\mathbf{y}^{(\mathrm{EV})}$, $\mathbf{y}^{(\mathrm{OD})}$ or $\mathbf{y}^{(1)}$. Furthermore, for $p_1 = P(r)$,*

$$\lim_{\Lambda \nearrow \mathbb{Z}^d} \mu_\Lambda(\,\cdot\,|\mathbf{y}^{(\mathrm{EV})}_{\Lambda^{\complement}}) = \mu^{(\mathrm{EV})}, \qquad \lim_{\Lambda \nearrow \mathbb{Z}^d} \mu_\Lambda(\,\cdot\,|\mathbf{y}^{(\mathrm{OD})}_{\Lambda^{\complement}}) = \mu^{(\mathrm{OD})} \tag{7}$$

and

$$\lim_{\Lambda\nearrow\mathbb{Z}^d}\mu_\Lambda(\,\cdot\,|\mathbf{y}^{(1)}_{\Lambda^\complement})=\mu^{(1)},\qquad \lim_{\Lambda\nearrow\mathbb{Z}^d}\mu_\Lambda(\,\cdot\,|\mathbf{y}^{(0)}_{\Lambda^\complement})=(\mu^{(1)}+\mu^{(\mathrm{EV})}+\mu^{(\mathrm{OD})})/3. \tag{8}$$

□

Pictorially, Theorem 1 shows a competition between different types in a high-density regime ($p_1+p_2=r$ close to 1). Namely, there is a curve $r\in(0,1)\mapsto(P(r),r-P(r))$ inside triangle $\mathbb{T}$ with the property that, for r close to 1, interval I_r (cf. (2)) is divided by the point $(P(r),r-P(r))$ into two halves. On one half, where $p_1>P(r)$, we have a single stable/dominant phase $\mu^{(1)}$. On the other half we have coexistence of two stable/dominant phases, $\mu^{(\mathrm{EV})}$ and $\mu^{(\mathrm{OD})}$ (a double co-existence.). Finally, at the meeting point where $p_1=P(r)$, $p_2=r-P(r)$, there are three phases co-existing, $\mu^{(1)}$, $\mu^{(\mathrm{EV})}$ and $\mu^{(\mathrm{OD})}$ (a triple co-existence).

It has to be said that the choice of the minimal distance collection $\mathbf{D}$ may result in different patterns of behavior. The above specification, with $D(1,1)=1$, $D(2,2)=2$, leads to a picture which is relatively well understood. In particular, empty boundary condition $\mathbf{y}_{\Lambda^\complement}$ (used in actual simulations) generate symmetric formulas in Eqns (6) and (8). Other choices of the self-exclusion minimal distances $D(1,1)$ and $D(2,2)$ would produce more complex patterns. This might lead to a slower convergence to a Markov chain steady-state distribution and result in a considerable instability of simulation results.

CDH1 Immunofluorescence

In cell co-culture experiments, cells were seeded in 24-well plates at 250 cells/mm^2 with glass coverslips. Each day, three coverslips were removed from the plate and cells were washed with PBS and fixed with cold methanol:acetone (1:1) solution for 15 minutes at 10 °C. After fixation, cells were briefly washed with PBS followed by antigen blocking with 5% bovine serum albumin (BSA) in PBS for 1 hour at room temperature. Cells were washed briefly in 1% BSA and incubated with 1:200 anti-CDH1 primary antibody (BD Transduction Laboratories, 610182) in 1% BSA solution for 1 hour at room temperature. After washing cells with incubation solution for 10 minutes three times at room temperature, cells were then labeled with 1:400 anti-mouse AlexaFluor546-conjugated antibody (Molecular Probes, A11003) and 50 µg/mL Hoechst 33258 stain (Molecular Probes, H3569) in 1% BSA for 2 hours at room temperature. After washing cells with incubation solution for 10 minutes three times at room temperature, slides were mounted with 1:1 glycerol:PBS solution and coverslips sealed.

Image Analysis

The 30 images for each day (10 from each coverslips) were acquired using E600 Nikon microscope under 20x objective with the respective fluorescent light filters for each marker. The difference in CDH1 label patterns was used to distinguish between HaCaT and SK-MEL-147 in co-culture images. Images have had the background noise subtracted and contrast enhanced in each channel. Colors were merged and cell count was estimated based on the pseudo-colored blue nuclei present in each image using ImageJ's Cell Counter plug-in [1]. The distances between cells were measured based on the centroid point of the selected cell nuclei.

Data Fitting

Cells counted in each image were analyzed using `R` software [2]. First, the data was read and each cell type was counted, resulting in the number of cells per image for cancer and normal cells. Averages of counts and standard deviation were calculated. The data was fitted and parameters of the logistic growth function (Eq. 9) estimated using the implemented `nls` function [3], where K is the carrying capacity (asymptote) given in the same units of the cell population P (cells/image), ρ is the proliferation rate (days^{-1}) and τ is the time t when half-asymptote is reached $\left(P(\tau)=\frac{K}{2}\right)$ [4].

$$P(t)=\frac{K}{1+e^{-\rho\cdot(t-\tau)}} \tag{9}$$

Below is presented the summary of the parameter values estimated for single cell culture proliferation of SK-MEL-147 and HaCaT cell lines.

```
Formula: SK147 population ~ K/(1 + exp(-rho * (time - tau)))

Parameters:
     Estimate Std. Error t value Pr(>|t|)
K   5043.5128   316.4690   15.94  < 2e-16 ***
rho    1.0462     0.1738    6.02 7.52e-08 ***
tau    4.8593     0.2126   22.86  < 2e-16 ***
---
Signif. codes:  0 ‘***’ 0.001 ‘**’ 0.01 ‘*’ 0.05 ‘.’ 0.1 ‘ ’ 1

Residual standard error: 778.4 on 69 degrees of freedom

Number of iterations to convergence: 9
Achieved convergence tolerance: 5.141e-06
```

```
Formula: HaCaT population ~ K/(1 + exp(-rho * (time - tau)))

Parameters:
     Estimate Std. Error t value Pr(>|t|)
K   1779.5603   130.4704  13.640  < 2e-16 ***
rho    1.1360     0.3978   2.856  0.00567 **
tau    2.5736     0.3345   7.695 7.22e-11 ***
---
Signif. codes:  0 ‘***’ 0.001 ‘**’ 0.01 ‘*’ 0.05 ‘.’ 0.1 ‘ ’ 1

Residual standard error: 638.7 on 69 degrees of freedom

Number of iterations to convergence: 6
Achieved convergence tolerance: 2.953e-06
```

Keratinocytes reach lower cell density than melanoma cells at confluence

To distinguish between keratinocytes and melanoma cell lines in co-culture experiments we marked cells with anti-CDH1 antibodies. The immortalized keratinocytes HaCaT cell line present a distinct membrane pattern of CDH1 under fluoresce microscopy (Fig. S1A) and the human metastatic melanoma SK-MEL-147 cell line present low expression of E-cadherin (CDH1) (Fig. S1B). To evaluate cell proliferation, cells were seeded in a 24-well plate at 250 cells/mm^2. Cell density was calculated daily from counts in Neubauer chamber. The maximal density of melanoma cells was 2.8 times of keratinocytes after 8 days in culture (Fig. S1C). This indicates that the apparent growth arrest of keratinocytes cells is associated with cell density and a degree in contact inhibition.

Cell Co-culture

For co-culture experiments, cells were seeded at a proportion of 10 keratinocytes for each melanoma cell. This ratio was selected to mimic the epidermal basal layer [5]. However, cells were seeded at the same proportion (1:1) resulted in the formation of a "normal" cell clusters surrounded by tumor cells after 5 days. This indicates that cancer cells will eventually occupy all the space available (Fig. S4). As we can see on panel presented on Fig. S2, the melanoma cell clusters sizes range from 1000 µm^2 to 5.5×10^4 µm^2 in area.

Clusters Circularity

There is a good agreement between the aspect ratios measured for median obtained from both simulation and experiments (Fig. 4F). However the same level of agreement is not observed for the

circularity (Fig. S3). The experimental images are in the 2D Cartesian domain. The simulations geometry is a square grid where the diamond shape is the equivalent of a circle and its circularity is $\pi/4$, that is, the shapes of the clusters are grid anisotropy dependent [6]. The surroundings of the domains are rough and that causes a difference between the circularity as measured from the clusters of our images and the expected circularity if shape of our clusters were an ellipse (experiments) or a diamond (simulations) [7].

Distance Distribution Analysis

The distribution of distances between cells was performed in selected images acquired in LSM 510 Meta confocal microscope (Carl Zeiss) under 20x objective. Cells were counted as previously described (See sections **Image Analysis** and **Data Fitting**) and the centroid position of each cell nucleus in an image was used as reference. The euclidean distance matrix was generated using the `dist` function and the distribution was calculated using `density` function. The distance distribution of keratinocytes assumes only distances that are found within the same distance as the melanoma cell clusters. The minimal distance is defined as the most frequent distance observed (mode) for each cell type in a given image. The mean minimal distance and standard deviation for each day was calculated. When confluence is reached, the average minimal distance between melanoma cells is reduced showing its lower exclusion diameter (Fig. S5A). An increase in the difference between the minimal distances of the two cell types indicates the clustering of melanoma cells (Fig. S5B).

Simulations

Additional simulation results are presented aiming to demonstrate that the cell type having the smallest exclusion diameters shall prevail on the spatial domain (Fig. S6). Here we kept the same values for all the remaining parameters of the model as our goal is to exemplify the importance of loss of contact inhibition for the prevalence of the tumor cells. On the left graph we have kept the exclusion diameters of the tumor cells to be smaller than for the healthy cells. We show that the tumor cells will prevail at the steady state regime. On the right graph we have inverted the exclusion diameters and made the healthy cells to have the smallest exclusion diameters and we show their prevalence at the steady state regime.

References

[1] Caroline A. Schneider, Wayne S. Rasband, and Kevin W. Eliceiri. NIH Image to ImageJ: 25 years of image analysis. *Nature Methods*, 9(7):671–675, July 2012.

[2] R Core Team. *R: A Language and Environment for Statistical Computing*. R Foundation for Statistical Computing, Vienna, Austria, 2017.

[3] John Fox, Sanford Weisberg, and John Fox. *An R companion to applied regression*. SAGE Publications, Thousand Oaks, Calif, 2nd ed edition, 2011. OCLC: ocn648922089.

[4] Sébastien Benzekry, Clare Lamont, Afshin Beheshti, Amanda Tracz, John M. L. Ebos, Lynn Hlatky, and Philip Hahnfeldt. Classical mathematical models for description and prediction of experimental tumor growth. *PLoS computational biology*, 10(8):e1003800, August 2014.

[5] Mirosława Cichorek, Małgorzata Wachulska, Aneta Stasiewicz, and Agata Tymińska. Skin melanocytes: biology and development. *Postepy Dermatol Alergol*, 30(1):30–41, 2013.

[6] M. Marek. Grid anisotropy reduction for simulation of growth processes with cellular automaton. *Physica D: Nonlinear Phenomena*, 253:73–84, 2013.

[7] Y. Takashimizu and M. Iiyoshi. New parameter of roundness r: circularity corrected by aspect ratio. *Progress in Earth and Planetary Science*, 3:2, 2016.

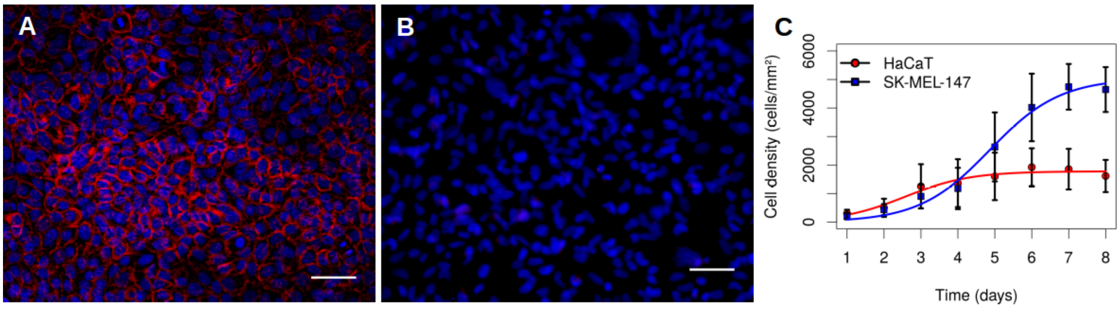

Figure S1: Keratinocytes reach lower cell density than melanoma cells at confluence. (**A**) and (**B**) Immunofluorescent staining of E-Cadherin (CDH1) on HaCaT and SK-MEL-147 cells, respectively. Different pattern on CDH1 staining was used as marker to count cells in co-culture experiments. Cells grown in DMEM (Gibco) were fixed with cold methanol:acetone. Then, cells were stained with mouse anti-E-cadherin and Alexa Fluor 546 Anti-mouse secondary antibody, (pseudo-colored in red). Nuclei staining was with DAPI (pseudo-colored blue). The images were captured on Nikon Eclipse E600 microscope and merged using ImageJ software. Scale bar: 50 µm. (**C**) Comparison of proliferation curves attained by HaCaT and SK-MEL-147 cell lines. Cells were seeded in 24-well plate at initial density of 250 cells/mm^2 and counted daily in Neubauer chamber. Points represents averages of three independent experiments in triplicates and bars standard deviations. Lines are the fitted data obtained from logistic growth model parameter estimation.

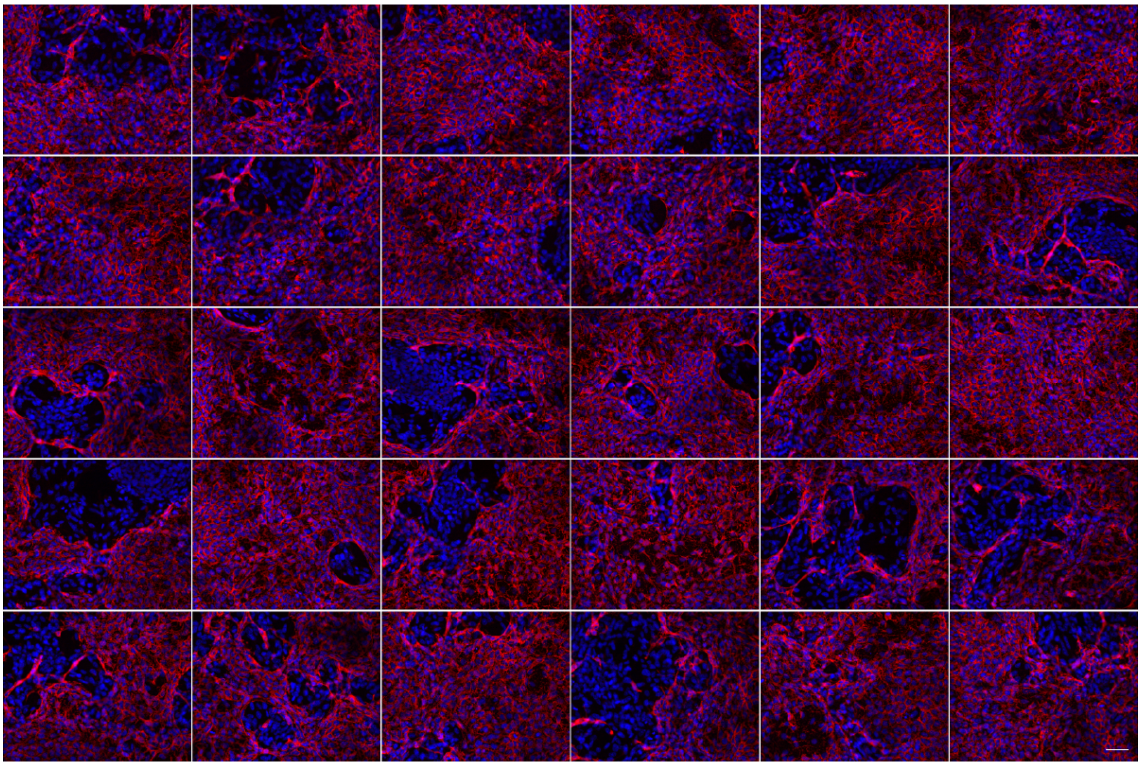

Figure S2: Panel of melanoma cell clusters surrounded by keratinocytes after 8 days in co-culture. Each image represents one field of view ($\sim$ 412 x 330 µm^2) from co-culture slides with cells fixed and stained for nuclei (pseudo-colored in blue) and CDH1 (pseudo-colored in red). Scale bar: 50 µm.

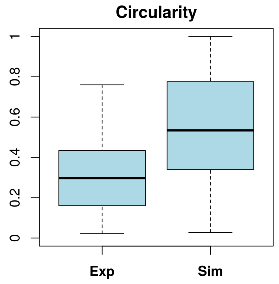

Figure S3: Clusters circularity. The difference in the circularity is due to the geometry of the simulations is a square grid where the diamond shape is the equivalent of a circle.

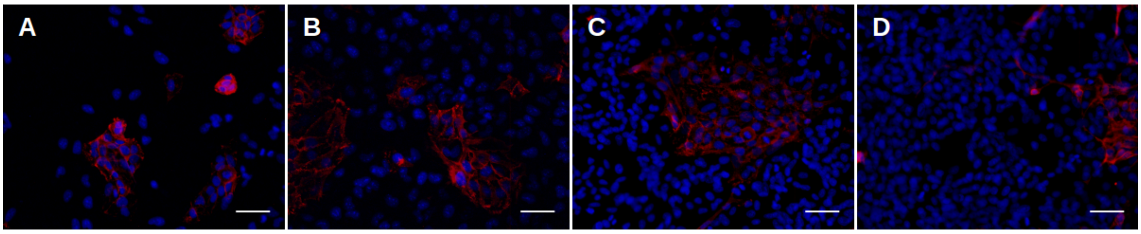

Figure S4: Co-culture of SK-MEL-147 and HaCaT cell lines. Cells were seeded at 1:1 proportion in 24-well plate with coverslips. Each image represents one field of view ($\sim$ 412 x 330 µm^2) from co-culture slides with cells fixed and stained for nuclei (pseudo-colored in blue) and CDH1 (pseudo-colored in red). Scale bar: 50 µm.

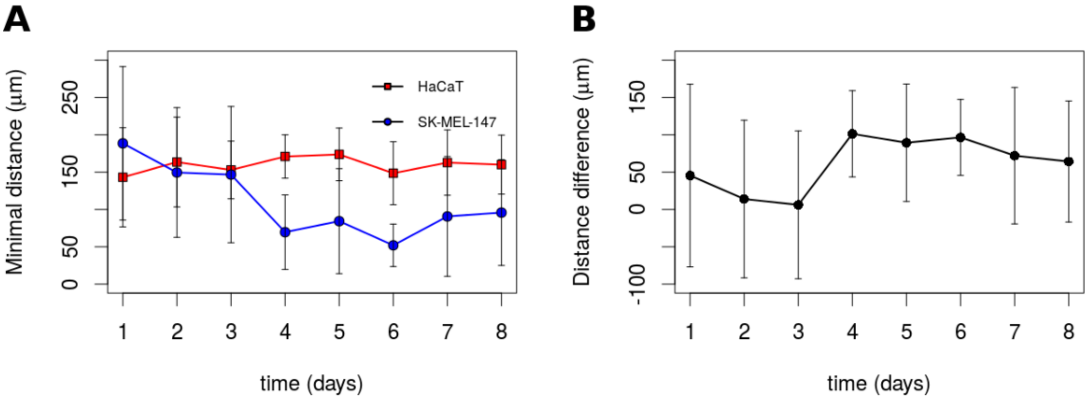

Figure S5: (**A**) The minimal distance observed for each day of co-culture experiment. SK-MEL-147 confluence shows a shorter distance than HaCaT cells. (**B**) The increasing distance between SK-MEL-147 and HaCaT cell lines indicates the greater tolerance and clustering pattern of cancer cells. Points indicates the averages and bars the standard deviation.

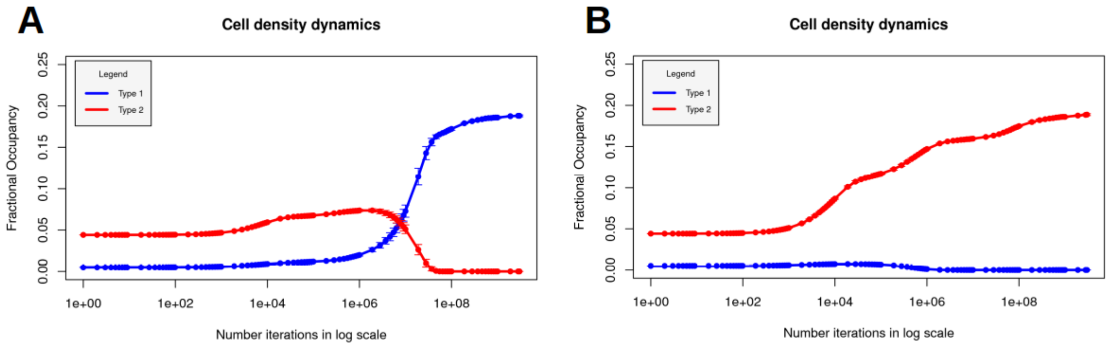

Figure S6: The density dynamics of the stochastic model for different sets of the exclusion diameters. The division rates b $\alpha_1 = \alpha_2 = 0.1$, the degradation rates $\rho_1 = \rho_2 = 0.01$ and the migration rates $\delta_1 = \delta_2 = 0.001$. The exclusion diameters were chosen to be $D(1,1) = 2$, $D(1,2) = D(2,1) = 4$, and $D(2,2) = 3$ on the left graph and right graph has $D(1,1) = 3$, $D(1,2) = D(2,1) = 4$, and $D(2,2) = 2$.
